# Ecological Study of Variability in the Relationship between Liver Cancer Mortality and Racial Residential Segregation

**DOI:** 10.3390/ijerph18189732

**Published:** 2021-09-15

**Authors:** Amin Bemanian, Laura D. Cassidy, Raphael Fraser, Purushottam W. Laud, Kia Saeian, Kirsten M. M. Beyer

**Affiliations:** 1 Pediatric Residency Program, University of Washington, P.O. Box 5371, OC.7.830, Seattle, WA 98105, USA; 2 Institute for Health and Equity, Medical College of Wisconsin, 8701 Watertown Plank Road, Milwaukee, WI 53226, USA; lcassidy@mcw.edu (L.D.C.); rfraser@mcw.edu (R.F.); laud@mcw.edu (P.W.L.); kbeyer@mcw.edu (K.M.M.B.); 3 Division of Gastroenterology and Hepatology, Medical College of Wisconsin, 8701 Watertown Plank Road, Milwaukee, WI 53226, USA; ksaeian@mcw.edu

**Keywords:** racial segregation, cancer epidemiology, structural racism, liver cancer

## Abstract

Racial segregation has been identified as a predictor for the burden of cancer in several different metropolitan areas across the United States. This ecological study tested relationships between racial segregation and liver cancer mortality across several different metropolitan statistical areas in Wisconsin. Tract-level liver cancer mortality rates were calculated using cases from 2003–2012. Hotspot analysis was conducted and segregation scores in high, low, and baseline mortality tracts were compared using ANOVA. Spatial regression analysis was done, controlling for socioeconomic advantage and rurality. Black isolation scores were significantly higher in high-mortality tracts compared to baseline and low-mortality tracts, but stratification by metropolitan areas found this relationship was driven by two of the five metropolitan areas. Hispanic isolation was predictive for higher mortality in regression analysis, but this effect was not found across all metropolitan areas. This study showed associations between liver cancer mortality and racial segregation but also found that this relationship was not generalizable to all metropolitan areas in the study area.

## 1. Introduction

Liver cancer is a major cause of cancer mortality in the United States. In 2019, there were estimated to be 42,030 newly diagnosed cases of liver cancer and 31,780 liver cancer deaths [1]. Furthermore, liver cancer has an exceptionally poor five-year survival rate, at 18%. Liver cancer is notable for having marked racial disparities in incidence and mortality. Based on 2018 US mortality rates, Black/African Americans (8.2 deaths per 100,000), Hispanics (9.4 per 100,000), Asian/Pacific Islanders (8.2 per 100,000), and American Indians/Alaskan Natives (10.8 per 100,000) have significantly higher mortality rates of liver cancer compared to non-Hispanic Whites (5.9 per 100,000) [2]. Furthermore, Black five-year liver cancer survival rates are known to be poorer compared to Whites (14% vs. 17%) [1]. Traditionally, these differences have been attributed to racial differences in the prevalence of various risk factors and precursor diseases associated with liver cancer (e.g., viral hepatitis) or due to differences in access to care [3,4,5].

Research on other cancers suggests that there is a significant role for the social environment in the development of cancer and its association with racial disparities in cancer outcomes. Ethnic enclaves have been shown to be associated with higher risk of infection-associated cancers, such as cervical and liver [6]. For breast cancer, racial segregation and housing discrimination have been associated with differences in survival rates [7,8,9,10]. Ethnic enclaves with lower socioeconomic status have also been associated with higher liver cancer incidence rates [11]. One study of liver cancer prevalence in New York City found that a ZIP-code-tabulated area (ZCTA) poverty rate was associated with the prevalence rate of liver cancer even after controlling for the hepatitis C and hepatitis B prevalence rates [12]. However, the literature on the effects of geography and place in liver cancer remains limited. Furthermore, most studies on liver cancer focus on racial disparities for Hispanics and Asians/Pacific Islanders, while Black disparities in liver cancer have been understudied [6].

Studies of racial segregation and cancer have often pooled together patients across multiple regions or metropolitan areas to assess if there is a relationship between segregation and cancer risk [10,13,14]. While this has the benefits of increasing sample size and including a diverse population, there is a risk that the included areas may be too heterogeneous. This is especially significant for studying racial segregation, which emerged and manifested in a variety of different ways throughout American history [15,16]. Furthermore, patterns of immigration in the latter half of the twentieth century have been highly variable depending on ethnicity and specific area of the country, and the health impacts of ethnic enclaves have been highly varied [17,18,19]. As a result, combining different metropolitan areas may risk obscuring real place-specific relationships.

In this paper, we investigate how liver cancer in major metropolitan areas in the state of Wisconsin is related to the racial, spatial, and socioeconomic distribution of its residents and how these relationships vary across the state. We focus on two specific questions. First, we seek to identify if there is a relationship between racial residential segregation and liver cancer mortality rates. This analysis is conducted by pooling Wisconsin’s five largest metropolitan statistical areas (MSA) that are fully enclosed within the state. Then, we seek to determine whether relationships between segregation and liver cancer are place-dependent. In order to answer this question, the analysis of segregation and mortality is stratified for each of these five metropolitan areas, and multivariate regression models are developed for the two largest Wisconsin metropolitan areas: Milwaukee– Waukesha–West Allis, WI MSA and Madison, WI MSA.

## 2. Materials and Methods

Tract-level liver cancer standardized mortality rates (SMRs) in Wisconsin were calculated. Mortality data were obtained from the Wisconsin Department of Health Statistics’ Vital Records Service for all cases of liver cancer (International Classification of Diseases (ICD) Version 10 Codes: C22.0–C22.9) from 1 January 2003 to 31 December 2012 in the state of Wisconsin. A total of 3204 liver cancer deaths were included in the analysis. Age-adjusted SMRs were calculated in a prior study using adaptive spatial filtering (ASF), which incorporates dynamic spatial filters to overcome issues with having a low number of cases and stabilizes the rates. [20,21] This results in a continuous surface, which was averaged per tract to estimate tract-level rates.

The study area included the five largest metropolitan areas in Wisconsin, excluding any MSAs that crossed state boundaries. These were the Milwaukee–Waukesha–West Allis MSA, the Madison MSA, the Appleton–Oshkosh–Neenah combined statistical area (CSA), the Green Bay MSA, and the Racine MSA. The Appleton–Oshkosh–Neenah CSA was used instead of the Appleton and Oshkosh–Neenah MSAs because Appleton and Neenah have overlapping municipalities, despite being in separate MSAs. Based on the 2010 US census, these five regions combined comprise 3,055,652 residents and 53.7% of Wisconsin residents [22].

Demographic data were obtained from the 2008–2012 five-year American Community Survey (ACS). Neighborhood socioeconomic status was calculated using an index of socioeconomic advantage (SEA) that has been previously used for a study of liver cancer mortality in Wisconsin [21]. This index is calculated from the percentage of tract residents who graduated with a bachelor’s degree or higher, the percentage of residents who were unemployed, and the tract’s median household income. Due to concern for instability in the estimates for the socioeconomic variables, SEA was not calculated in tracts if the standard errors for any of the three ACS variables were outliers (>1.5 the interquartile range). Positive SEA scores indicate higher socioeconomic status relative to the statewide average. Rurality was calculated as the proportion of rural census blocks within a census tract, according to the 2010 US Census urban/rural designations [22]. Tract-level racial segregation was measured using local Black and Hispanic isolation scores, which were calculated using the Local Exposure/Isolation (LEx/Is) index [9]. This metric measures how much a subarea’s racial/ethnic demographics deviate from the expected case that all racial/ethnic groups are equally distributed across a region. Positive local isolation scores indicate the subarea has a higher proportion of that specific racial/ethnic group than the proportion in the region. LEx/Is scores are scaled using a base-10 logit function. The advantage of the LEx/Is scores is that by normalizing against an expected case of homogenous mixing, we can calculate the index for any metropolitan area and do normalized comparisons across regions.

Clusters of high and low mortality (i.e., hot- and coldspots) were identified using the Getis–Ord G_i_ statistic [23]. A threshold of *p* < 0.05 was used to identify high and low clusters. The calculation of SMRs included the entire state in order to have stable enough populations to conduct the (ASF) technique in the outer tracts of MSAs. As a result, mortality cluster identification involved the entire state as well.

Liver cancer mortality and demographic covariates (SEAs, rurality, and isolation scores) across the five MSAs were initially compared using chi-squared and Kruskal–Wallis analysis of variance (ANOVA). We then investigated the bivariate relationship between which mortality clusters tracts were included, and the various demographic covariates, using ANOVA with an α of 0.05 selected for statistical significance. These relationships were tested first for all the MSAs pooled together and then stratified by MSA. The MSA-specific tests were corrected for multiple comparisons using the Benjamini–Hochberg method, given the risk of false discovery by repeating the testing for each MSA.

Log-linear regression models to assess the relationship between the demographic covariates and liver cancer SMRs were also developed. These regressions incorporated spatially adjusted errors in order to correct for significant spatial autocorrelation [24]. The first regression pooled all the tracts across the five metropolitan areas and used fixed effects to control for baseline differences in SMR across the metropolitan areas. Stratified regressions for the Milwaukee and Madison MSAs were also calculated to assess whether or not the relationships were modified by metropolitan area. Milwaukee and Madison were selected for individual regression analyses because they were large enough MSAs to not be concerned with overfitting (N of 429 and 131 tracts, respectively). All analysis was conducted using the R statistical programming language (3.60, R Foundation for Statistical Computing, Vienna, Austria) [25].

## 3. Results

Comparisons of the five MSAs are shown in Table 1. The average liver cancer SMR and proportion of tracts in a mortality hot- or coldspot is significantly different across the MSAs. Milwaukee had the highest mean SMR (1.311) of all MSAs and the largest proportion of tracts in a high-mortality cluster (45.2%). The Appleton MSA had significantly lower mortality than the other MSAs, with a mean SMR of 0.773, no high-mortality cluster tracts, and with 95.7% of its tracts in low-mortality clusters. SEA significantly differed across MSAs, with Madison having the highest mean SEA and Milwaukee having the lowest. Milwaukee was significantly more urban than all other MSAs.

**Figure 1:**
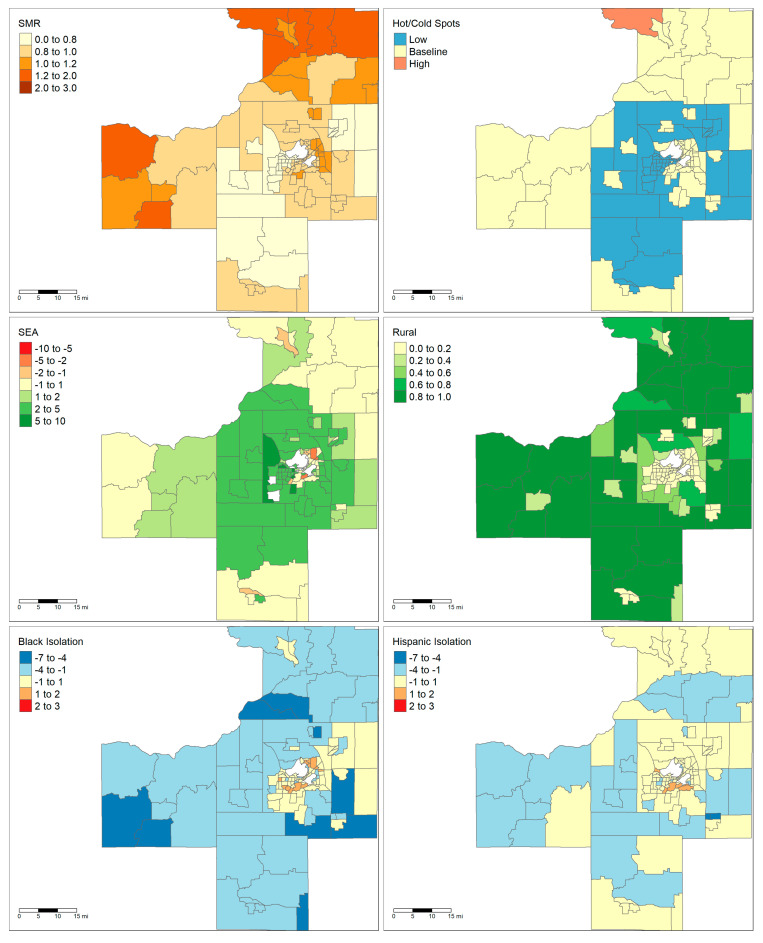
Maps of Madison, WI MSA. Upper left: liver cancer SMRs. Upper right: high- and low-mortality clusters of liver cancer. Middle left: socioeconomic advantage index with white tracts indicating areas whose ACS errors were outliers. Middle right: rurality. Lower left: Black local isolation scores. Lower right: Hispanic local isolation scores. Abbreviations: WI: Wisconsin, SMR: Standardized Mortality Rate, ACS: American Community Survey.

**Figure 2:**
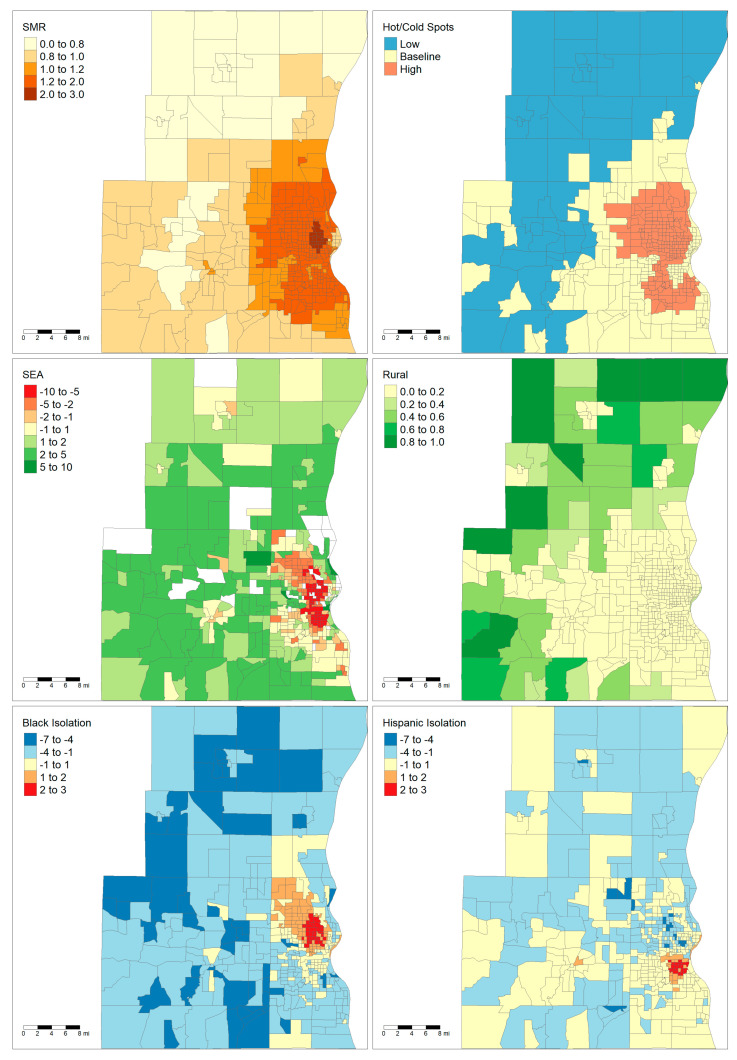
Maps of Milwaukee–West Allis–Wauwatosa, WI MSA. Upper left: liver cancer SMRs. Upper right: high- and low-mortality clusters of liver cancer. Middle left: socioeconomic advantage index with white tracts indicating areas whose ACS errors were outliers. Middle right: rurality. Lower left: Black local isolation scores. Lower right: Hispanic local isolation scores. Abbreviations: WI: Wisconsin, SMR: Standardized Mortality Rate, ACS: American Community Survey.

The two largest areas, Milwaukee and Madison, are notable for having very different demographic and socioeconomic characteristics. Maps showing the demographics of these two areas are shown in Figure 1 and Figure 2. The majority of the land area of Madison is rural except for the City of Madison in the center and several population centers in the neighboring counties. By contrast, Milwaukee has very low rurality throughout the entire metropolitan area. Madison only has one tract that falls within a high cancer mortality cluster, while Milwaukee has a large cluster of high mortality in the central city. Finally, the maps show that there are clear foci of Black and Hispanic isolation for the Milwaukee MSA but not in the Madison MSA.

**Figure 3:**
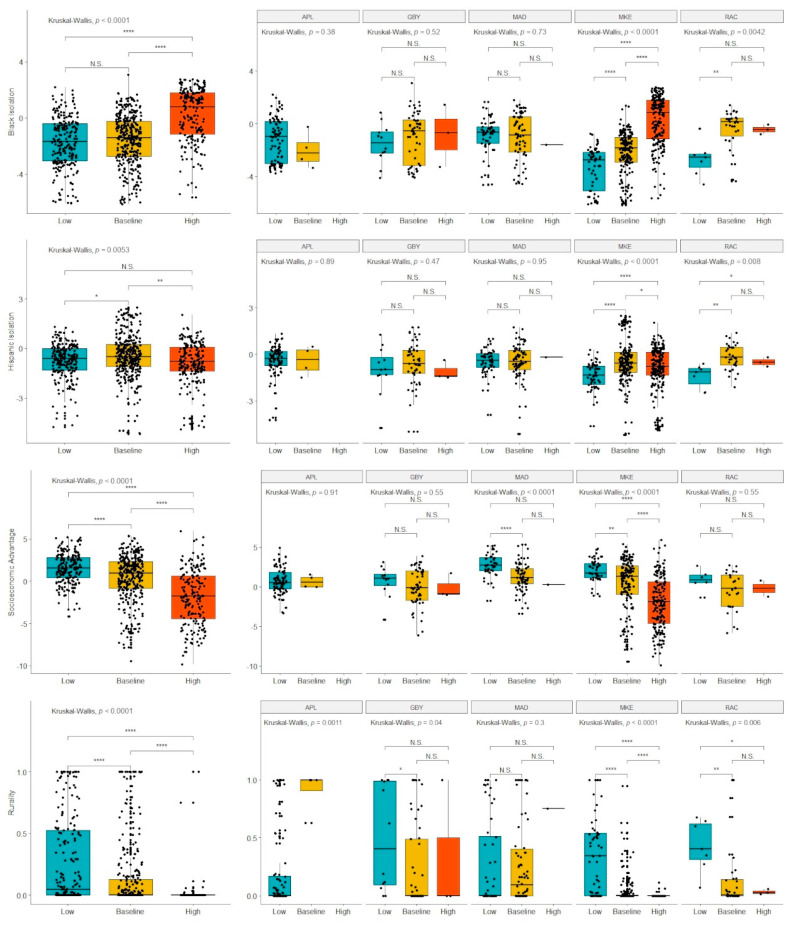
Boxplots comparing segregation and other demographic variables within high-, baseline-, and low-mortality tracts. The left-most column indicates comparison pooling all five MSAs (APL: Appleton, GBY: Green Bay, MAD: Madison, MKE: Milwaukee, RAC: Racine). The columns on the right-hand side show comparisons within each individual MSA. Statistical significance was assessed using the Kruskal–Wallis test. The *p*-values were adjusted for multiple comparisons with Benjamini–Hochberg correction for MSA-specific testing. * indicates *p* < 0.05, ** indicates *p* < 0.01, *** indicates *p* < 0.001, **** indicates *p* < 0.0001. Abbreviation: N.S.: Not Significant.

Boxplots comparing mortality coldspots, mortality hotspots, and baseline mortality tracts are shown in Figure 3. These analyses were conducted across all five regions and then stratified for each region. Since Appleton had no mortality hotspots, its analysis was a simple non-parametric comparison of the means with no further multiple comparisons testing. Across all regions, higher Black isolation, lower SEA, and lower rurality were associated with increased likelihood of a tract being in a high-mortality cluster and decreased likelihood of being in a low-mortality cluster. Hispanic isolation was found to be the highest in the baseline tracts with both high- and low-mortality cluster tracts having significantly lower Hispanic isolation scores. Stratifying the analysis, we find that only Milwaukee and Racine have evidence of a significant relationship between Black isolation and mortality cluster status. For Milwaukee, there is a significant, monotonic relationship from low- to high-mortality clusters, but Racine showed no significant difference between average and high-mortality clusters. For socioeconomic status, only Milwaukee and Madison showed a significant relationship with cluster status. For rurality, Milwaukee, Racine, and Green Bay showed evidence of the trend found in the pooled analysis. However, in the greater Appleton metropolitan area, the low-mortality cluster was associated with lower rurality. Notably, all significant relationships in the pooled analysis were also significant in the Milwaukee analysis, and only two significant comparisons in Milwaukee (low- vs. baseline-mortality tracts for Black isolation and low- vs. high-mortality tracts for Hispanic isolation) were not significant in the pooled analysis.

The log-linear regression models to estimate tract liver cancer SMRs are shown in Table 2. This includes one model that pools all five areas and two specific models focusing on the Madison and Milwaukee MSAs. All models were adjusted using spatial errors, and the pooled analysis included hierarchical variables to control for baseline SMR differences for each MSA. Non-spatially adjusted regression was tested as well, but the residuals were significantly spatially autocorrelated (testing with Moran’s I: *p* < 0.001). Racine had a significantly higher baseline SMR with a rate ratio (RR) of 1.581 relative to Appleton. Hispanic isolation was found to be the only other significant predictor of SMR, with higher Hispanic isolation associated with increased SMR. The stratified analysis of Milwaukee also found a similar relationship between Hispanic isolation and liver cancer SMR. No predictors were significant in the Madison model.

## 4. Discussion

This paper sought to identify what relationships exist between liver cancer mortality and segregation, and how these relationships vary across different metropolitan areas. Our analysis focused on five different metropolitan areas, which, despite being in the same state, are very different in demographics. Milwaukee and Racine tend to be more urban and have lower average socioeconomic status compared to the other regions studied. Furthermore, they had the highest average levels of liver cancer mortality. Given these differences in baseline characteristics, it is not entirely surprising that relationships between demographic variables and cancer mortality varied significantly across MSAs. Comparing the pooled hotspot analyses to the stratified analyses reveals that the pooled findings are primarily being driven by relationships found in the Milwaukee MSA. The population of the Milwaukee MSA is greater than the other four areas combined (1,555,954 vs. 1,499,778) and it comprises 56.2% of the number of tracts included in the analysis [22]. This highlights how one study area can skew the findings of a combined analysis.

The mortality hotspot analyses did show evidence of potential relationships between liver cancer and racial segregation. In the Milwaukee MSA, Black isolation was significantly higher in mortality hotspots and significantly lower in mortality coldspots than in baseline tracts. The only other MSA which had similar findings was Racine. Milwaukee (16.8%) and Racine (11.1%) do have a substantially larger proportion of Black residents compared to the other MSAs (Madison: 4.6%, Green Bay: 1.8%, Appleton–Oshkosh: 1.3%). Interestingly, while Milwaukee has the highest degree of segregation as measured by the Black–White dissimilarity score (0.783), Racine’s level of segregation (0.475) falls in the same range as the other areas (Madison: 0.478, Green Bay: 0.480, Appleton–Oshkosh: 0.417) [26]. Despite the clear relationship between Black isolation and mortality in the hotspot analysis in Milwaukee, the spatially adjusted regression found no relationship. However, the spatially adjusted regression is complicated by the fact that the ASF method used to calculate liver cancer mortality uses cases in neighboring tracts in order to stabilize the rates [20,21]. Therefore, the liver cancer SMRs are intrinsically spatially dependent. Furthermore, Black isolation is heavily clustered within a single region in Milwaukee (Figure 2). It is hard to determine if there is a consistent relationship between Black isolation and liver cancer mortality or if the relationship is specific to the place itself.

The relationship between Hispanic isolation and liver cancer mortality is more ambiguous. Hotspot analysis found significant differences for Hispanic isolation in Milwaukee, Racine, and the pooled analysis, and the regression analysis showed that Hispanic isolation was associated with increased liver cancer mortality even with spatial adjustment. However, deeper inspection of the hotspot analysis shows that both mortality hotspots and coldspots have significantly lower Hispanic isolation than the baseline tracts in Milwaukee. The lack of a clear exposure–response relationship for Hispanic isolation makes inferring a meaningful relationship difficult. The pooled hotspot analyses found monotonic exposure–response relationships for socioeconomic advantage and rurality, but neither of these predictors were significant in any of the spatial regression analyses. Given that socioeconomic status and racial demographics of neighborhoods are frequently related, there was concern multicollinearity could be affecting the regression analyses [14,27]. However, multicollinearity was tested using variance inflation factors, and all combinations were below 2.0, suggesting weak evidence for multicollinearity.

It is important to note that these differences in how the metropolitan areas are racially structured stem from historical political and economic pressures. Milwaukee was historically a major manufacturing and industrial hub that had a large influx of African-Americans during the Great Migration in the early 20th century and later experienced a significant exodus of Whites from the city center to the suburbs [15,28]. These migration patterns resulted in Milwaukee being more racially diverse than the rest of the state (69.0% White, 16.4% Black, 9.5% Hispanic). In a 2010 study of segregation across the 100 largest metropolitan areas in the United States, Milwaukee ranked first in Black–White dissimilarity and ninth in Hispanic–White dissimilarity [29]. In contrast, Madison (MSA 2010 population: 568,593) is the state capital and its economy has centered around the state government and the University of Wisconsin, so it has not experienced the same economic pressures as Milwaukee. Madison is less racially diverse than Milwaukee (83.7% White, 4.5% Black, 5.4% Hispanic), and it was ranked 71st for Black–White dissimilarity and 65th for Hispanic–White dissimilarity [29]. The differences in how these metropolitan populations formed could also impact how health inequities between groups formed.

The relationship between rurality and liver cancer mortality was highly specific to the metropolitan area, with more rural tracts more likely to be liver cancer coldspots in Milwaukee, Racine, and Green Bay while less rural tracts were more likely to be coldspots in the Appleton–Oshkosh area. The finding that rurality was associated with decreased mortality in the pooled analysis was surprising given how previous studies have shown that rural counties have higher rates of risk factors associated with liver cancer such as obesity and smoking [30]. Some of this discrepancy may be due to how rurality was defined in this study. The rurality score represents the proportion of blocks within the census tracts that are classified as “rural” by the US Census Bureau. Per the Census Bureau, an urban area is defined as any area of census blocks or tracts that has a core population of at least 2500 people, and any outlying non-residential areas attached to it [31]. Any census block which falls outside of urban areas is classified as “rural.” Comparing Figure 1 and Figure 2 reveals that the distribution of rural blocks is very different in Madison versus Milwaukee. Many of Milwaukee’s highly “rural” tracts are immediately adjacent or one neighbor removed from a predominately urban tract. This suggests that these tracts are simply less developed and most likely have similar access to healthcare resources as their more urban neighbors. While only a small fraction of Milwaukee’s tracts have greater than 80% rurality, the majority of Madison’s tracts by land area have that level of rurality. Furthermore, Madison’s outlying counties only have small islands of urbanicity when compared to the large suburban sprawl of Milwaukee. This highlights how “rural” tracts were heterogenous depending on the study area.

This study is limited by its cross-sectional, ecological design, which prevents causal inference. The outcomes and determinants of health under study were defined at the level of the census tract, while liver cancer occurs on the level of an individual. Furthermore, these models can only examine the relationship between the social determinants and liver cancer at the point of death. Given the typically long latency from the initial development of liver disease to liver cancer and death [32,33], this is an incomplete assessment of the role social determinants may play in affecting liver cancer disparities. The relative rarity of liver cancer is another challenge. We used adaptive spatial filtering to map liver cancer mortality in Wisconsin, because directly adjusted mortality rates cannot be stably calculated for such a small area as a census tract. This approach can result in a smoothing of the mortality rate, which could lead tracts with extreme rates to be regressed to the means of their neighbors. Nevertheless, this is a necessary adjustment to be able to do an analysis on a sub-county level.

An additional limitation is the issue of survey error in the socioeconomic variables used to calculate SEA. While the American Community Survey is a major asset for population health and demographics research, the stability of the estimates for small areas such as tracts has been raised as a potential concern [34]. By removing the tracts with extreme levels of error, we were able to mitigate some of the risk of unstable estimates while continuing to have a high-resolution analysis. Finally, it is important to note that death-certificate data have a risk of imperfect coverage of all liver cancer cases. Previous analysis of death certificates found there is an 87.1% detection rate of liver and intrahepatic bile cancers for ICD-9 coded certificates and a 76.9% detection rate for ICD-10 coded certificates [35]. While there is a risk that missing liver cancer cases may not be truly randomly distributed across Wisconsin’s population, death certificates provide the highest quality data we have available to conduct these analyses. Despite these limitations, we believe this study provides important insight in identifying which factors may be influencing liver cancer disparities and highlights the importance of centering the role of “place” when looking at heterogenous populations.

## 5. Conclusions

This study investigated the relationship between tract-level liver cancer mortality with racial segregation, socioeconomic status, and rurality. Additionally, this study highlighted how these relationships can vary across different metropolitan areas even if they are within the same state. Black local isolation was shown to be a predictor of liver cancer mortality in Milwaukee and Racine, but not in other MSAs. Socioeconomic advantage was associated with lower levels of liver cancer mortality. The variable nature of these relationships across metropolitan areas highlights the importance of place in analyses of cancer disparities. Given how the findings of this study differ from previous ones in describing how ethnic enclaves and racial segregation are associated with liver cancer, further work should be done to characterize the distribution of liver cancer across a diverse set of geographic regions. More research needs to be done on the level of the individual to identify how racial segregation affects patients with liver disease and liver cancer. In terms of developing public health interventions to combat liver cancer and reduce disparities, this study suggests that it may be more effective to target specific geographic areas and communities with place-dependent interventions rather than deploying interventions over wide areas.

## Figures and Tables

**Table 1:** Comparisons of the Wisconsin MSAs included in the study. The top portion of the table compares the number of tracts which are mortality hotspots and coldspots across the different MSAs. The *p*-value reported is for chi-squared testing. The bottom portion of the table shows mean and standard deviations of the continuous variables in this study: liver cancer SMR, SEA, localized Black and Hispanic isolation, and the proportion of rural tracts. The *p*-values reported for these variables are based on Kruskal–Wallis testing across MSAs. Bolded *p*-values indicate statistical significance of *p* < 0.05. Abbreviations: MSA: Metropolitan Statistical Area, SMR: Standardized Mortality Ratio, SEA: Socioeconomic Advantage, SD: Standard Deviation.

Variable	Appleton, WI		Green Bay, WI		Madison, WI		Milwaukee, WI		Racine, WI		Significance
	N		N		N		N		N		
**All Tracts**	92		68		131		429		44		
Mortality Clusters	**n**	%	**n**	%	**n**	%	**n**	%	**n**	%	*p*
High	0	0.0	3	4.4	1	0.7	194	45.2	3	6.8	<0.001
Low	88	95.7	12	17.6	56	42.7	56	13.1	7	15.9	
**Variables**	**Mean**	**SD**	**Mean**	**SD**	**Mean**	**SD**	**Mean**	**SD**	**Mean**	**SD**	***p***
SMR	0.773	0.054	1.036	0.210	0.897	0.144	1.311	0.463	1.104	0.188	<**0.001**
SEA	0.713	1.664	0.067	2.289	1.857	1.803	−0.478	3.417	−0.245	2.284	<**0.001**
Black Isolation	−1.243	1.679	−1.088	1.842	−0.938	1.579	−1.254	2.259	−0.725	1.599	0.198
Hispanic Isolation	−0.391	0.984	−0.731	1.447	−0.466	1.032	−0.744	1.352	−0.406	0.911	<**0.001**
Rurality	0.214	0.349	0.288	0.396	0.279	0.377	0.065	0.183	0.171	0.260	<**0.001**

**Table 2:** Spatially adjusted log-linear regression models of liver cancer standardized mortality ratios. The first set of columns is the result of the pooled analysis across all five MSAs, which includes the fixed-effect coefficient for each MSA. The second and third set of columns are results of analysis limited to tracts within the Milwaukee and Madison MSAs respectively. Bolded coefficients are statistically significant predictors for *p* < 0.05. Abbreviations: RR: rate ratio for each covariate, CI: Confidence Interval, NA: Not applicable.

Covariate	All MSAs		Milwaukee MSA		Madison MSA	
	RR	95% CI	RR	95% CI	RR	95% CI
SEA	0.997	0.992–1.001	0.994	0.988–1.000	0.997	0.987–1.008
Black Isolation	1.001	0.996–1.006	1.004	0.996–1.012	0.996	0.983–1.008
Hispanic Isolation	**1.007**	**1.001–1.014**	**1.015**	**1.005–1.024**	1.006	0.990–1.022
Rurality	1.005	0.967–1.045	0.985	0.906–1.069	0.999	0.939–1.064
**MSA Effect**	**RR**	**95% CI**				
Appleton	1 (Control)	NA	-	-	-	-
Green Bay	1.019	0.879–1.182	-	-	-	-
Madison	1.182	0.699–1.737	-	-	-	-
Milwaukee	1.357	0.956–1.927	-	-	-	-
Racine	**1.581**	**1.088–2.298**	-	-	-	-
